# Activation of PKR Causes Amyloid ß-Peptide Accumulation via De-Repression of BACE1 Expression

**DOI:** 10.1371/journal.pone.0021456

**Published:** 2011-06-28

**Authors:** Gerard ILL-Raga, Ernest Palomer, Matthew A. Wozniak, Eva Ramos-Fernández, Mònica Bosch-Morató, Marta Tajes, Francesc X. Guix, José J. Galán, Jordi Clarimón, Carmen Antúnez, Luis M. Real, Mercé Boada, Ruth F. Itzhaki, César Fandos, Francisco J. Muñoz

**Affiliations:** 1 Laboratory of Molecular Physiology and Channelopathies, Department of Experimental and Health Sciences, Universitat Pompeu Fabra (UPF), Barcelona, Catalonia, Spain; 2 University of Manchester, Manchester, United Kingdom; 3 NeoCodex, Sevilla, Spain; 4 Alzheimer Laboratory, Neurology Department, Hospital de la Santa Creu i Sant Pau, Centro de Investigación Biomédica en Red sobre Enfermedades Neurodegenerativas (CIBERNED), Barcelona, Spain; 5 Fundación Alzheimur, Unidad de Demencias, Hospital Virgen de la Arrixaca de Murcia, Murcia, Spain; 6 Memory Clinic of ACE Foundation, Catalan Institute of Applied Neurosciences, Barcelona, Spain; 7 Neurology Department, Hospital G. Universitari Vall d'Hebron, Barcelona, Spain; University of Texas M. D. Anderson Cancer Center, United States of America

## Abstract

BACE1 is a key enzyme involved in the production of amyloid ß-peptide (Aß) in Alzheimer's disease (AD) brains. Normally, its expression is constitutively inhibited due to the presence of the 5′untranslated region (5′UTR) in the BACE1 promoter. BACE1 expression is activated by phosphorylation of the eukaryotic initiation factor (eIF)2-alpha, which reverses the inhibitory effect exerted by BACE1 5′UTR. There are four kinases associated with different types of stress that could phosphorylate eIF2-alpha. Here we focus on the double-stranded (ds) RNA-activated protein kinase (PKR). PKR is activated during viral infection, including that of herpes simplex virus type 1 (HSV1), a virus suggested to be implicated in the development of AD, acting when present in brains of carriers of the type 4 allele of the apolipoprotein E gene. HSV1 is a dsDNA virus but it has genes on both strands of the genome, and from these genes complementary RNA molecules are transcribed. These could activate BACE1 expression by the PKR pathway. Here we demonstrate in HSV1-infected neuroblastoma cells, and in peripheral nervous tissue from HSV1-infected mice, that HSV1 activates PKR. Cloning BACE1 5′UTR upstream of a luciferase (luc) gene confirmed its inhibitory effect, which can be prevented by salubrinal, an inhibitor of the eIF2-alpha phosphatase PP1c. Treatment with the dsRNA analog poly (I∶C) mimicked the stimulatory effect exerted by salubrinal over BACE1 translation in the 5′UTR-luc construct and increased Aß production in HEK-APPsw cells. Summarizing, our data suggest that PKR activated in brain by HSV1 could play an important role in the development of AD.

## Introduction

Alzheimer's disease (AD) is a neurodegenerative disorder that affects 18 million people worldwide. Sufferers experience severe memory deficits and cognitive decline, and their brains are characterized by two pathological features – neurofibrillary tangles and senile plaques. The former consist mainly of abnormally phosphorylated forms of the microtubule-associated protein tau, and the latter of amyloid ß-protein (Aß) [1], a peptide generated by the enzymatic cleavage of an integral membrane glycoprotein called amyloid precursor protein (APP). One of the enzymes involved in APP cleavage is BACE1 (ß-site APP Cleaving Enzyme) [2], [3], [4], [5], [6], a single pass transmembrane peptidase belonging to the aspartyl protease family [7]. BACE1 expression is elevated in AD patients but the causes are unknown [8], [9], [10]. Interestingly, BACE1 protein levels are elevated in AD brains whereas BACE1 mRNA levels remain unaltered [9], [10], [8]. Furthermore, despite the fact that stimuli such as oxidative stress [11], [12] or hypoxia [13], [14] activate BACE1 transcription, upregulating BACE1 mRNA levels, BACE1 translation is basally repressed by its 5′untranslated region (5′UTR) [15], [16], [17], [18]. The 5′UTR of BACE1 is 470 nucleotides long, rich in GC (∼70%) and it contains four upstream initiation codons (uAUGs). All four uAUGs, but especially the second one, are responsible for the translational arrest that the 5′UTR exerts over the BACE1 open reading frame (ORF) [16].

The eukaryotic initiation factor 2-alpha (eIF2-alpha) is a GTP-binding protein that catalyses the loading of the first met-tRNA into the ribosome to initiate protein translation [19]. When eIF2-alpha is phosphorylated at Ser51, translation initiation is arrested, a scenario required in the event of cellular compromise such as glucose deprivation, ER stress or viral infection [20]. However, there is a specific subset of mRNAs whose translation is activated in response to eIF2-alpha phosphorylation [21], [22]. A recent study shows that BACE1 belongs to this subset of mRNAs, demonstrating that eIF2-alpha phosphorylation activates BACE1 translation [23]. Consequently, factors that promote phosphorylation of eIF2α at Ser51 might contribute to elevated BACE1 expression, leading to an increase in cleavage of APP and Aβ accumulation.

There are four kinases that are capable of phosphorylating eIF2α at Ser51: double-stranded RNA-activated protein kinase (PKR), PKR-like ER-localized eIF2α kinase (PERK), heme-regulated inhibitor kinase (HRI; present in erythrocytes) and general control non-derepressible 2 kinase (GCN2; activated by amino acid deprivation) [24]. PKR has an N-terminal domain that behaves as a molecular sensor for any double stranded RNA (dsRNA) formed during the replication of viral genomes [25]. Molecular recognition of dsRNA at the PKR N-terminal domain induces its autophosphorylation at multiple sites but especially at Thr451 and Thr446, the former being critical for the kinase activity [26], catalysing eIF2-alpha phosphorylation at Ser51. Therefore, PKR is a defensive viral sensor that shuts off global protein synthesis to prevent viral protein replication within the host cell [27]. However, this defensive cellular mechanism might have a negative side-effect, as BACE1 translation is activated in response to eIF2-alpha phosphorylation [23], causing breakdown of APP to Aβ. Thus PKR activation might be an important factor in AD, and in fact, a potentially functional variation of EIF2AK2 gene (the gene coding for PKR) has been associated with AD [28] and activated PKR has been found in the brains of AD sufferers [29].

Many viruses cause PKR activation but only one of these has been consistently linked to AD – herpes simplex virus type 1 (HSV1). HSV1 is a neurotropic virus that infects most humans, usually during infancy. Once a person is infected, the virus persists lifelong in the peripheral nervous system. HSV1 is the cause of several diseases including herpes labialis, genital herpes and a severe, but rare, brain disorder called herpes simplex encephalitis (HSE). HSV1 was originally proposed as a factor in AD because the brain regions that are affected in HSE are the same as those in AD – the frontal and temporal cortices [30]. Subsequent work has shown that HSV1 is present in the brains of elderly humans [31] and that it confers risk of AD in subjects who possess a specific genetic factor: the type 4 allele of the apolipoprotein E gene (APOE-ε4) [32]. Recently, the virus was shown to cause Aβ accumulation [33] and abnormal tau phosphorylation [34], and to increase the levels of the associated enzymes, including BACE1 [33]. Also, HSV1 DNA was found to be very specifically associated with amyloid plaques in AD brains [34].

The mechanism by which HSV1 induces BACE1 expression is unknown. However, it is a plausible hypothesis that PKR activation and subsequent eIF2-alpha phosphorylation activate BACE1 translation in HSV1-infected neurons. In the present work we investigated this possibility. Firstly, we present evidence that HSV1 infection activates PKR in neuroblastoma cells and in dorsal root ganglion (DRG) from HSV1-infected mice. To provide a mechanistic insight into the way PKR induces BACE1 translation, we cloned BACE1 5′UTR and inserted it upstream of a luciferase (luc) gene. As expected, BACE1 5′UTR repressed the reporter signal. Salubrinal (Sal003), an inhibitor of the eIF2-alpha phosphatase PP1c, de-repressed the reporter signal in the 5′UTR-luc construct, demonstrating a positive effect exerted by eIF2-alpha phosphorylation over the 5′UTR-luc construct. Synthetic polyinosinic–polycytidylic acid (poly [I∶C]), which is an analog of dsRNA [35], [36], resulted in *in vitro* PKR activation and produced a recovery in 5′UTR-luc reporter signal comparable to that obtained by Sal003. Furthermore, a specific PKR inhibitor [37] reversed the rise in reporter signal induced by poly (I∶C), thereby indicating that PKR triggers BACE1 translation.

Previous studies reported PKR activation in neurons from AD brains [38], [29]. We confirmed these findings in brain sections that were previously tested for HSV1 infection [34]. Interestingly, activated PKR was only present in AD neurones although HSV1 DNA was present in the tissue from a non-demented subject as well.

## Materials and Methods

### Ethics statement

The procedure to dissect mouse embryos followed the E.U. guidelines for animal experimentation and it was approved by the Ethics Committee for Animal Experimentation of the Institut Municipal d'Investigacions Mèdiques (IMIM)-Universitat Pompeu Fabra (UPF) (Approval ID: JMC-07-1001P1-PML). Finally, human brain sample study was carried out with the ethical permission obtained from Tameside & Glossop Local Research Ethics Committee (UK; Approval ID: 05/Q1402/37). Written informed consent was obtained for all study participants.

### Reagents

Synthetic dsRNA polyinosinic:polycytidylic acid (poly (I∶C)) (Sigma) was purchased from Sigma-Aldrich (St. Louis, MO, USA). An imidazolo-oxindole compound that acts as a potent ATP-binding site directed inhibitor of PKR [37] was purchased from Calbiochem (San Diego, CA, USA). All media and additives for cell culture were purchased from Gibco (Carlsbad, CA, USA).

### Cell cultures

Human neuroblastoma cells (SH-SY5Y) were grown with F-12 (Ham) supplemented with 15% fetal bovine serum (FBS) and antibiotics (100 units/mL penicillin and 100 µg/mL streptomycin). HEK cells overexpressing the Swedish mutation of APP (HEK-APPsw) (kindly provided by Dr. B. de Strooper; K.U. Leuven, Belgium), PERK knock-out mouse embryonic fibroblast (MEF) cells (kindly provided by Dr. D. Cavener; Pennsylvania State University, USA) and HeLa cells were grown with Dulbecco's modified Eagle's medium (DMEM) supplemented with 10% FBS and antibiotics (100 units/mL penicillin and 100 µg/mL streptomycin). Cells were incubated at 37°C in a humidified atmosphere of 5% CO_2_.

### Mouse embryo hippocampal cell cultures

Hippocampal cells were isolated from 18-day-old OF1 mouse embryos. Hippocampi were aseptically dissected and trypsinized. Cells were seeded in phenol-red-free DMEM plus 10% horse serum on to 1% poly-L-Lysine coated coverslips (5×10^4^ cells/cover). After 120 min, medium was removed and neurobasal medium was added containing 1% B27 supplement (Gibco BRL) plus antibiotics. Cultured hippocampal cells were used for the experiments on day 7 when they were considered to be mature neurons [40].

### Human brain samples

Serial temporal lobe sections from an AD patient and an age-matched control were kindly provided by the South West Dementia Brain Bank, Bristol, UK. The individuals analysed were a 78 years old control female carrying the APOE genotype E3E3, and a 75 years old AD female carrying the APOE genotype E3E4. A previous study [34] carried out on sequential sections of the same samples revealed that HSV1 DNA was present in both.

### Mouse dorsal ganglion samples

Sections of DRG from HSV1-infected mice were kindly provided by Dr Stacey Efstathiou, University of Cambridge, UK. Five 8–9 week old BALB/c mice were infected with 10^6^ plaque forming units (pfu) of HSV1 strain SC16 by ear scarification in the left ear pinna. Five days after infection the animals were killed and the CII, CIII and CIV cervical DRG were pooled from the five mice. The contralateral sensory ganglia from the same animals were used as control, uninfected ganglia.

### Protein identification by Western Blot

Cells were lysed on ice with a solution containing 1 M Tris-HCl, 1% Nonidet P-40, 150 mM NaCl, 5 mM EDTA, 1 mM sodium orthovanadate, 1 mM dithiotreitol, pH 7.4 and a protease inhibitor cocktail (Roche, Basel, Switzerland). Protein concentration was determined by Bio-Rad protein assay. Protein samples were electrophoretically resolved within 10% Tris-HCl gels run at 150 V for 1 h and afterwards transferred to nitrocellulose membranes using iBlot Gel Transfer System (Invitrogen). Membranes were blocked in Tween 20-Tris buffer solution (TTBS: 0.1% v/v Tween 20, 100 mM Tris-HCl, 150 mM NaCl, pH 7.5), containing 5% milk, and incubated overnight at 4°C with 1∶500 rabbit anti-PKR (Abcam), 1∶1000 rabbit anti-phospho-PKR (Thr446; p-PKR) (Abcam), 1∶500 rabbit anti phospho-eIF2-alpha Ser51; p-eIF2-alpha) (Cell signalling), 1∶1000 rabbit anti-BACE1 (Chemicon International), 1∶1000 mouse anti-eIF2-alpha (Cell signalling), 1∶1000 mouse anti-6E10 (Covance; Atom), 1∶1000 mouse anti-APP (N-terminal) (Oncogene; Calbiochem), 1∶1000 rabbit anti-cleaved Notch 1 (Val1744) (Cell signalling) and 1∶7000 mouse anti-tubulin (Sigma), respectively. Primary antibodies (Abs) were diluted either in 5% skimmed milk-TTBS (anti-PKR, anti-p-PKR, anti-BACE1, anti-6E10, anti-APP and anti-tubulin) or in 5% bovine serum albumin in TTBS (anti-p-eIF2-alpha, anti-eIF2-alpha and anti-Notch). Peroxidase-conjugated donkey anti-rabbit and anti-mouse (Amersham Bioscience, Buckinghamshire, U.K.) were used as secondary Abs at 1∶5000 for 1 h at room temperature (RT). Bands were visualized with Super Signal (Pierce, Rockford, IL, USA) and Amersham Bioscience Hyperfilm ECL kit.

### Infection of SH-SY5Y cells with HSV1

HSV1 (strain SC16) stocks were prepared as described previously [41]. SH-SY5Y cells were seeded at a concentration of 10.74 million cells per flask (T75) and incubated overnight. Prior to infection, growth medium was discarded and cells were then washed briefly with 10 mL of PBS at 37°C. HSV1 was added to 1 mL of 0.5% FBS supplemented growth medium at 3 pfu/cell. During the 1 h of incubation, the HSV1-inoculated medium was gently distributed homogeneously on the flask surface. This procedure was repeated four times, every 15 min. Afterwards, the inoculated medium was removed, and 10 mL of fresh growth medium plus 0.5% FBS was added, followed by incubation for 24 h. For luciferase assays of BACE1 5′UTR, cells could not be infected with HSV1, as infected cells do not survive long enough for transfection procedures to be performed. For that reason, we used poly (I∶C) as an accepted model for viral infection [35], [36], [42].

### Poly (I∶C) treatment in PERK knock-out fibroblasts

MEFs PERK^−/−^ were seeded in 6-well plates and grown in normal growth medium (DMEM+10% FBS+Antibiotics) until they reached a confluence of ∼80%. To stimulate the cells with poly (I∶C) the growing medium was replaced for growth medium containing 5 µg/mL poly (I∶C). Control cells were not exposed to poly (I∶C) and they were left in growth medium during the experiment. Cells were treated with poly (I∶C) for 3 h or 6 h. Cells were washed twice in PBS, lysed and proteins were extracted.

### Poly (I∶C) treatments to study gamma-secretase activity

HEK-APPsw were seeded in 6-well plates and grown in normal growth medium (DMEM+10% FBS+Antibiotics) until they reached a confluence of ∼80%. The cells were incubated with 10 µM of DAPT (gamma-secretase inhibitor) for 24 h. Then cells were treated with 5 µg/mL poly (I∶C) for 3 h. Cells were washed twice in PBS, lysed and proteins were extracted. C99, total APP, Notch Intracellular C-Domain (NICD) and soluble APPalpha (sAPPalpha) were studied by western blot as indicated above.

### Immunocytochemistry, immunohistochemstry and immunofluorescence

For immunocytochemistry, SH-SY5Y cells were grown on aminopropylethoxysilane (APES)-coated glass slides and infected with HSV1 (one pfu/cell). Slides were fixed for 10 min in 4% formalin and 10% acetic acid in PBS, washed and left overnight in 70% ethanol. Afterwards, slides were washed twice, for 5 min, in Tris-Buffer saline (TBS) and then rinsed in 20% (v/v) acetic acid for 45 s to inhibit endogenous alkaline phosphatase activity. Next, slides were washed twice in TBS containing 0.025% (v/v) Triton X-100 and blocked in 10% (w/v) skimmed milk in TBS for 1 h at RT on a shaker. After two further washes in TBS, primary Abs were applied in 1% (w/v) skimmed milk in TBS and incubated overnight in an hydration chamber at the following dilutions: 1∶100 rabbit anti-p-eIF2-alpha Ser51) (Cell signalling), 1∶100 rabbit anti-p-PKR (Thr446; Abcam) and 1∶100 rabbit anti-BACE1 (Abcam). Slides were rinsed in TBS containing 0.025% Triton X-100 and treated for 2 h with a biotinylated secondary Ab (Abcam) diluted in TBS plus 1% skimmed milk. Slides were again washed twice in TBS and then treated with streptavidin-alkaline phosphatase conjugate (Sigma) for 30 min. After two further washes in TBS, substrate (5-bromo-4-chloro-3-indolyl phosphate/nitro blue tetrazolium) was added and slides incubated for 30–120 min before rinsing, mounting and UV curing.

For immunohistochemistry, human brain temporal lobe sections and mouse DRG sections were dehydrated by treating successively with 70%, 90% and absolute ethanol, dewaxed in 100% xylene and rehydrated by using the ethanol series in reverse. Next, in order to expose epitopes, slides were rinsed three times in PBS for five minutes per wash, placed in an acidic buffer (sodium citrate 10 mM, pH 6) and autoclaved for one cycle in a Prestige Medical. Afterwards, slides were rinsed 3 times in TBS and the procedure continued as described above from the blocking of endogenous alkaline phosphatase activity step.

For immunofluorescence, hippocampal primary cultures were fixed with 4% paraformaldehyde (PFA) after treatment with poly (I∶C) at a final concentration of 5 µg/mL for 3 and 6 h. Afterwards, cells were permeabilized with 0.1% Triton X-100 and subsequently incubated for 2 h at RT in a hydration chamber with 1∶100 rabbit anti BACE1 Ab (Chemicon International), followed by incubation with 1∶700 Alexa Fluor 488 goat anti-rabbit polyclonal Ab for 1 h at RT. Finally, nuclei were stained with TO-PRO 3 iodide (Sigma). Coverslips were mounted and digital images were taken with a Leica TCS SP confocal microscope and analysed with Leica confocal software (Heidelberg, Germany).

For immunofluorescence of brain temporal lobe sections, the samples were dewaxed and subjected to an epitope exposure procedure as previously described. Then, sections were blocked in 1% skimmed milk-TBS for 1 h at RT. Both primary Abs, rabbit phospho-PKR(T446)(Abcam) and mouse BACE1 (Millipore), were incubated simultaneously in a solution of 1% skimmed milk-TBS at a dilution of 1∶50. Primary Abs were incubated overnight in a humidified chamber at 4°C. Then, slides were washed thrice in PBS-Triton (0,025%) and two fluorescent secondary Abs (rabbit Alexa-488 and mouse Alexa-555) were incubated in the dark for 1 h at a dilution of 1∶700. Afterwards, slides were washed and incubated for 10 min with a solution of TO-PRO3 iodide (diluted 1∶1000 in distilled water) for nuclear staining. Finally, slides were rinsed in distilled water, coverslips were mounted in mowiol and examined by confocal microscopy.

### Cloning of BACE1 5′-untranslated region

Total RNA was extracted from SH-SY5Y cells, and one-step RT-PCR was carried out using a kit (Qiagen) with primers designed to amplify BACE1 5′UTR: 5′-GAAGCTTACAAGTCTTTCCGCCTCCCC-3′, 5′-GAAGCTTGGTGGGCCCCGGCCTTC-3′. PCR product, a single band matching the molecular weight of BACE1 5′UTR (∼500 nt), was isolated and purified from an agarose gel using the Ilustra™ GFX™ PCR DNA and Gel Band Purification kit (GE Healthcare) and stored at −20°C for further uses. The 5′UTR DNA fragment was then inserted into the HindIII site of a modified pGL4.10[luc2]vector from Promega containing the CMV promoter cloned at BglII and HindIII sites).

### Transient DNA transfection of HeLa cells and Luciferase assay

HeLa cells were seeded in 96-well plates at a density of 9000 cells per well and grown for 12 h with DMEM plus 10% FBS. Afterwards, a total of 250 ng of DNA was transfected into each well, adjusting to the following conditions: 250 ng of pcDNA3 plasmid as blanks, 25 ng of Renilla+25 ng of CMV-Luciferase Vector+200 ng of pcDNA3 as controls, and finally 25 ng of Renilla+25 ng of BACE1-5′UTR CMV-Luciferase construct+200 ng of pcDNA3 as test samples. Cells were transfected using JetPEI transfection reagent (PolyPlus) for 2 h. Afterwards, medium was changed and cells were incubated for 10 h to allow sufficient gene expression. Then, the wells were preincubated with PKR inhibitor (Calbiochem) in DMEM supplemented with 10% FBS at a final concentration of 0.3 µM for 1 h. Next, medium containing PKR inhibitor was withdrawn, cells were washed once with PBS and replaced with DMEM plus 1% FBS, with or without poly (I∶C) at a final concentration of 5 µg/mL for 3 h. Luciferase and Renila activities were measured by using the Dual-Glo™ Luciferase Assay System (Promega) following the manufacturer's instructions, and luminescence was read using a luminometer (Fluostar OPTIMA, BMG labtech).

### Aß measurement

HEK-APPsw cells were grown and the growth medium was replaced for experimental medium: phenol-red free DMEM containing 4.5 g/L D-glucose, L-glutamine, HEPES (25 mM), antibiotics (100 units/mL penicillin and 10^−6^ µg/mL streptomycin) and supplemented with 0.2% FBS. Cells were treated with poly (I∶C) or untreated (controls). 800 µL of medium was collected in each experimental condition after 3 hr, and centrifuged at 13,000 rpm for 5 min to eliminate cellular debris. Solid-phase sandwich ELISA kits containing two highly specific Abs for detection of the Aß peptides were used following manufacturer's instructions to measure human A
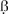
(1–40) and (1–42) (IBL Codes 27714 and 27712 respectively). Briefly, samples were added to ELISA plates pre-coated with anti-human A
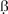
(35–40) mouse IgG affinity purified monoclonal Ab or anti-human A
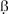
(38–42) rabbit IgG affinity purified Ab. After overnight incubation and washing, labelled Ab solution (horseradish peroxidase-conjugated anti-human A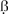 (N) rabbit IgG affinity purified Ab or horseradish peroxidase-conjugated anti-human A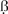 (N) rabbit IgG Fab affinity purified) was added for 1 h at RT and then washed. The chromagen (tetramethyl benzidine solution) was added and incubated for 30 min. The absorbance at 450 nm was determined for each sample.

### Cell viability assay

HeLa cells were seeded in 96-well plates in serum- and phenol red-free medium at a density of 1×10^3^ cells/100 µL/well. Cells were treated with the PKR inhibitor (Calbiochem) at a concentration of 0.3 µM during 1 h. Alternatively, cells were preincubated with the PKR inhibitor in the previously mentioned conditions of time and concentration and subsequently treated with poly (I∶C) (Sigma) at a final concentration of 5 µg/mL. Cells were placed in the incubator (37°C, 5% CO_2_) during the incubation time.

Cell viability was measured by 3-(4,5-dimethylthiazol-2-yl)-2,5-diphenyltetrazolium bromide (MTT) reduction. Briefly, 11 µL of MTT stock solution (5 mg/mL) were added and after 2 h the reaction was stopped with 120 µL of DMSO. MTT reduction was determined in a spectrophotometer at 540 and 650 nm. Control cell values were taken as 100%.

## Results

### HSV1 infection activates PKR

**Figure 1:**
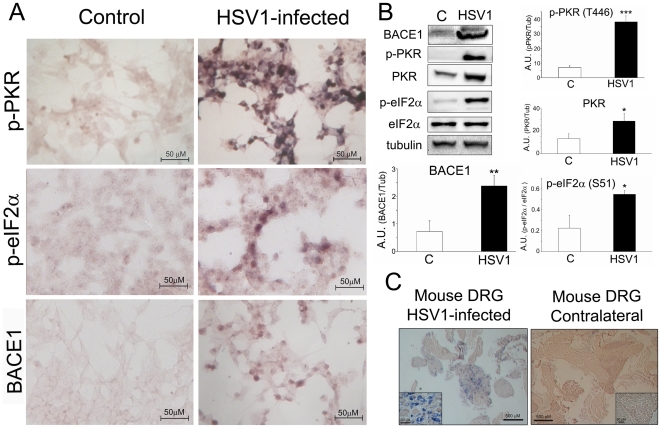
HSV1 infection activates PKR. SH-SY5Y cells were infected with HSV1 (1 pfu/cell, 24 h) or were not infected (controls). Immunocytochemistry analysis was carried with the following Abs: anti-p-PKR, anti-p-eIF2-alpha and anti-BACE1 (A). Protein extracts of SH-SY5Y cells infected with HSV1 (3 pfu/cell, 24 h) and uninfected cells (controls) were analysed by Western blotting using the following Abs: anti-BACE1, anti-p-PKR, anti-PKR, anti-p-eIF2-alpha, anti- eIF2-alpha and anti-tubulin. Bands were quantified by densitometric analysis. Results are expressed as the mean ± SEM of 3–4 independent experiments. * p<0.05, ** p<0.01, *** p<0.0005 by Student's *t* test (B). Sections from mouse dorsal ganglion root (DRG) were obtained from HSV1-infected mice. Contralateral uninfected ganglia were used as controls. Immunohistochemistry analysis was carried out to detect p-PKR (C).

Previous research has shown that infection with HSV1 leads to activation of PKR which, in turn, causes phosphorylation of eIF2α and consequently triggers a shut-off of global protein synthesis [43]. We confirmed this finding in neuroblastoma cells (SH-SY5Y) infected with HSV1 for 24 h (Figs. 1A and 1B). Immunocytochemistry analysis showed a striking rise in PKR activation (phosphorylated at Thr446) in HSV1-infected cells (Fig. 1A, upper panels) that was accompanied by a rise in the signal corresponding to p-eIF2-alpha (middle panels) and BACE1 (lower panels). Western blot analysis of HSV1-infected neuroblastoma cells revealed a prominent band for activated PKR (p-PKR) that was absent in the control uninfected cells (p<0.0005; Fig. 1B) even when non-phosphorylated PKR was also present in control cells. Interestingly, non-phosphorylated PKR levels were also raised when cells were infected with HSV-1 (p<0.05; Fig. 1B). Western blotting confirmed that p-eIF2-alpha levels are larger in HSV1-infected than in uninfected cells (p<0.0005; Fig. 1B). Non-phosphorylated eIF2-alpha was used as control demonstrating that total expression of eIF2-alpha was not affected by the virus. To further study PKR activation by HSV1, we analysed peripheral nervous tissue from mice infected with HSV1 (Fig. 1C). Consistent with the results obtained in neuroblastoma cells, DRG infected with HSV1 showed a striking activation of PKR whereas no signal for active PKR appeared on the contralateral uninfected DRG (Fig. 1C).

Activated PKR phosphorylates eIF2-alpha at Ser51 enhances BACE1 translation [23]. Thus, HSV1 infection might be predicted to cause an increase in BACE1 protein via PKR and eIF2α phosphorylation. Indeed, previous work has shown that BACE1 is increased in HSV1-infected cells [33].

### Poly (I∶C) induces PKR activation, BACE1 upregulation and Aß production in cultured cells

**Figure 2:**
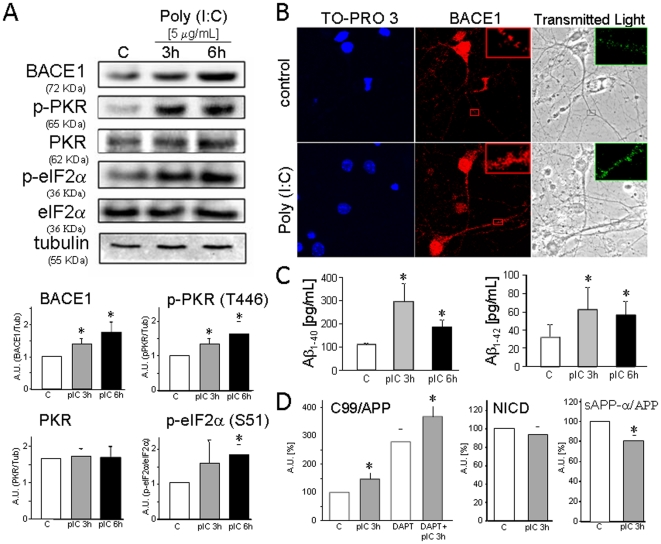
Poly (I∶C) induces PKR activation, upregulates BACE1 and promotes Aß production. The viral dsRNA analog polyinosinic:polycytidylic acid [poly (I∶C)] activates PKR in MEF PERK^−/−^ cells. Protein extracts obtained after 3 and 6 h of poly (I∶C) treatment were analysed by Western blotting using anti-BACE1, anti-phospho-PKR(T446), anti-PKR, anti-p-eIF2-alpha, anti-eIF2-alpha and anti-tubulin Abs. Bands were quantified by densitometric analysis. Results are expressed as the mean ± SEM of 3 independent experiments. * p<0.05 by Student's *t* test (A). Mature hippocampal neurons were challenged with poly (I∶C) for 3 h and analysed by immunofluorescence to detect BACE1 expression levels (red staining). BACE1 expression in neurites after 6 h of treatment (green staining) is shown in the insets in the transmitted light images (B). Poly (I∶C) triggers Aß_1–40_ and Aß_1–42_ production in HEK-APPsw cells. HEK-APPsw cells were challenged with poly (I∶C) at a concentration of 5 µg/mL. Then after 3 h and 6 h the media were collected and Aß_1–40_ and Aß_1–42_ levels were determined. Data are mean ± SEM of 5–12 independent experiments. *p<0.05 by Student's *t* test (C). Study of gamma-secretase activity in HEK-APPsw treated with poly (I∶C) at 3 h. Ratio of C99/total APP, NICD and ratio of sAPP-alpha/total APP were studied in protein extracts analyzed by Western Blotting. Bands were quantified by densitometric analysis. Y axis: arbitrary units. Results are expressed as the mean ± SEM of 3 independent experiments. * p<0.05 by Student's *t* test (D).

We used poly (I∶C), a synthetic viral dsRNA [35], [36], [42], to promote PKR activation *in vitro*. As shown in Fig. 2A, poly (I∶C) treatment (5 µg/mL) induces the active form of PKR (phosphorylated at Thr446) in PERK knock-out fibroblasts (p<0.05). Interestingly, BACE1 protein becomes rapidly upregulated in response to the initial rise of p-PKR (Thr446) obtained after 3 h of stimulation with poly (I∶C) (p<0.05; Fig. 2A) and it is maintained at 6 h (p<0.05; Fig. 2A). The same effect occurs in primary hippocampal cultures, in which high expression of BACE1, localized in the neurite network of hippocampal neurons, is observed after 3 h (and 6 h) of incubation with poly (I∶C) (Fig. 2B). Consistently, a significant phosphorylation of eIF2-alpha was observed after poly (I∶C) treatment (p<0.05; Fig. 2A) due to PKR activation. These results suggest that the increases in p-eIF2α and BACE-1 we observe are not due to PERK activation.

The prominent BACE1 up regulation in response to the viral dsRNA mimetic poly (I∶C) (Figs. 2A and 2B) led us to ask whether poly (I∶C) could also trigger Aß production *in vitro*. We found that poly (I∶C) triggers both Aß_1–40_ and Aß_1–42_ production (Fig. 2C) in HEK-APPsw cells. HEK-APPsw cells have a double mutation (Lys to Asn at residue 595 plus Met to Leu at position 596), which makes APP more prone to cleavage by BACE1, thus producing early AD onset [44]. HEK-APPsw treated with poly (I∶C) showed a dramatic increase in Aß_1–40_ at 3 h and 6 h (p<0.05), which was three- and two-fold the control value respectively (Fig. 2C). The pattern for Aß_1–42_ (Fig. 2C) was similar to that obtained with Aß_1–40_. Thus at 3 h and 6 h after poly (I∶C) treatment, Aß_1–42_ increases to two-fold the control value (p<0.05).

To confirm that the effect of poly (I∶C) was due to the up-regulation of BACE1 and not due to the up-regulation of gamma-secretase, we analyzed the activity of this complex. C99 is the C-terminal APP fragment containing the full Aß sequence after APP cleavage by BACE1. C99 is then cleaved by gamma-secretase to produce Aβ [7]. As expected, we found an increase in the C99/APP ratio (p<0.05) when cells HEK-APPsw were treated with poly (I∶C), and even when cells were treated with an inhibitor of gamma-secretase (DAPT; p<0.05) (Fig. 2D). Furthermore, we studied the levels of cleaved Notch (Notch Intracellular C-Domain; NICD), since Notch is one of the major substrates for gamma-secretase [7]. Our results did not show any variation in NICD production (Fig. 2D). Finally, we analyzed the levels of soluble APP-alpha (sAPP-alpha), which is a product of the non-amyloidogenic APP cleavage by alpha-secretases [7]. We observed a decrease of sAPP-alpha total APP ratio (p<0.05) in cells treated with poly (I∶C) (Fig. 2D), suggesting an increase in the amyloidogenic APP cleavage that fits with a BACE1 up-regulation.

### BACE1 translation is activated by a mechanism dependent on PKR activation and eIF2-alpha phosphorylation

**Figure 3:**
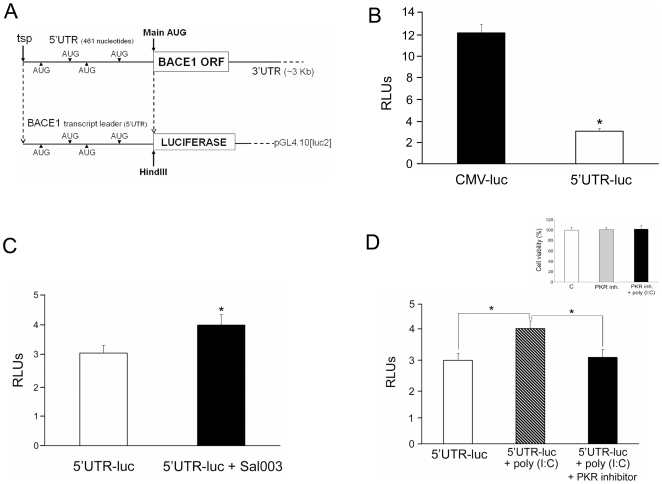
Poly (I∶C) induces BACE1 translation in a mechanism depending on PKR-catalysed eIF2-alpha phosphorylation. BACE1 5′UTR fragment was inserted into the HindIII site of a luciferase reporter construct (5′UTR-luc). The plasmids bear the strong cytomegalovirus promoter (CMV), which allows sufficient reporter gene expression for luciferase determinations. HeLa cells were transfected with 5′UTR-luc or empty plasmids (named as CMV) for luciferase reporter studies (A). 5′UTR-luc repressed translation in comparison with the empty 5′UTR-free luciferase construct (B). Inhibition of eIF2-alpha phosphatase PP1c by Sal003 de-repressed the reporter signal elicited by 5′UTR-luc (C). Stimulation (3 h) with poly (I∶C) also de-repressed the reporter signal yielded by 5′UTR-luc. This effect was abolished when cells were preincubated with a specific and potent PKR inhibitor that acts via the PKR ATP-binding site. Data are mean ± SEM of 3 independent experiments performed in triplicate. *p<0.05 by Student's *t* test (D). Incubation with the specific PKR inhibitor -in the presence/absence of poly (I∶C)- did not result in a decrease in cell viability as shown by an MTT assay. Data are mean ± SEM of 3 independent experiments performed in triplicate (Inset in panel D).

BACE1 translation is inhibited under basal conditions by its 5′UTR [15], [16], [17], [18], but this is reversed in response to eIF2-alpha phosphorylation (ser51) [23]. We cloned BACE1 5′UTR and inserted it upstream of a reporter luciferase gene (5′UTR-luc) (Fig. 3A). We used this 5′UTR-luc construct for transfection of HeLa cells. As expected, the basal effect of BACE1 5′UTR was to repress translation (Fig. 3B). The high reporter signal obtained with a strong cytomegalovirus promoter (CMV-luc) is reduced about three-fold when BACE1 5′UTR is inserted upstream of the luciferase gene (5′UTR-luc) without deleting the CMV promoter (Fig. 3B). Sal003, an inhibitor of eIF2-alpha phosphatase PP1c, de-repressed the reporter signal in 5′UTR-luc constructs, indicating that eIF2-alpha phosphorylation activates BACE1 translation, overcoming the repressor effect imposed by its 5′UTR (Fig. 3C). Interestingly, poly (I∶C) treatment resulted in the same effect as that obtained with Sal003 (Fig. 3D), lifting the repression of the 5′UTR-luc reporter signal with a detectable increase in luciferase signal. Importantly, this increase in luciferase signal obtained with poly (I∶C) treatment was fully reversed when cells were previously incubated with a specific imidazolo-oxindole PKR inhibitor [37] (Fig. 3D).

A possible cytotoxic effect of the inhibitor or poly (I∶C) treatments that could affect the obtained data was disproved since there were no changes in cell viability, as measured by MTT reduction assay (inset Fig. 3D).

### PKR activation in human brains

**Figure 4:**
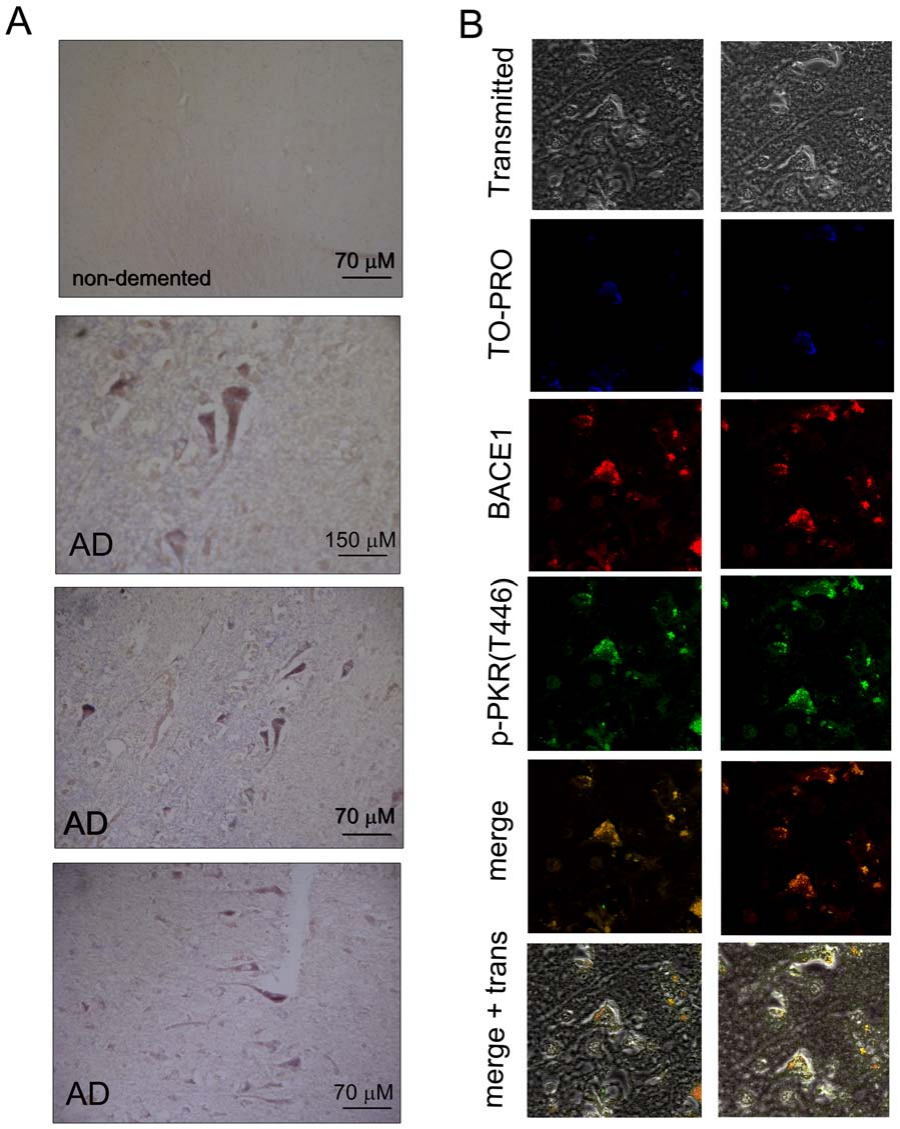
Presence of activated PKR in human brain tissue. The presence of activated PKR -autophosphorylated at Thr446- was studied in human brain sections (temporal lobe) from one non-demented control and one AD patient by immunohistochemistry (A). Colocalization of BACE1 and p-PKR(T446) in neurons from an AD patient. All brain sections analysed were positive for HSV1 infection as previously reported [39] (B).

Previous studies have documented PKR activation in AD brains [38], [29]. To confirm these findings, we chose brain sections from an AD patient and a non-demented individual both of whom were infected with HSV1 [45]. Interestingly, PKR activation was only detected in the AD brain tissue (Fig. 4A). HSV1 has its own mechanisms to circumvent host defensive mechanisms. One of these viral strategies consists of avoiding PKR activation by binding dsRNA species to viral proteins [46]. However, this system might be working deficiently in AD individuals, thereby accounting for their striking activation of PKR, due to PKR polymorphisms as suggested by Bullido et al. [47]. Consistently, PKR activation co-localizes with BACE1 expression in slides from AD brains, as we have observed by immunofluorescence analysis (Fig. 4B).

## Discussion

In the present work we provide experimental data supporting the proposition that HSV1 is a major risk factor for AD [32], [28], [33], acting perhaps by increasing the Aβ level via an increased expression of BACE1 [33]. We suggest that the BACE1 increase occurs through activation of PKR, leading to BACE1 up-regulation through eIF2-alpha phosphorylation-mediated de-repression of BACE1 translation. The p-eIF2-alpha inhibition of translation affects most mRNAs except those bearing a 5′UTR with specific features: long (>200 nucleotides), rich in GC content (∼70%) and containing upstream initiation codons; these mRNAs, including BACE1 mRNA, will be more efficiently translated into proteins during infection as a consequence of eIF2-alpha phosphorylation. Therefore, the increase in BACE1 expression characteristic of AD could be partially due to the translational derepression of BACE1 mRNA exerted by HSV1. However, when the translational arrest in global protein synthesis is sustained over time, the infected cell undergoes programmed cell death [48], [49], [50]. Interestingly, in recent years an involvement of PKR in neurodegeneration including AD has been reported [51], which proposes a proapoptotic role for activated PKR [29], [38], [52]. Intermittent activation of PKR during the lifetime of neurons by transient activation of latent HSV1 could contribute to increased APP amyloidogenic cleavage. Other causes of BACE1 activation by eIF2α phosphorylation have been investigated. A recent study [23] showed that cellular stress induced by glucose deprivation leads to BACE1 translation via eIF2α phosphorylation, in an effect mediated by PERK, one of the four kinases that are able to phosphorylate eIF2α. [24]; however, this effect was mediated by PERK and not PKR as in our studies. Indeed, we have found that poly (I∶C), a viral dsRNA analogue, is able to induce PKR activation by phosphorylation at Thr446 [53] and, consistently, its substrate, eIF2-alpha is phosphorylated at Ser51 [54]. This suggests that the effects of HSV1 are not specific to this virus and that other viruses may cause elevated BACE1 levels. However, it should be stressed that HSV1 is uniquely able to cause such changes as it is present in a high proportion of elderly brains. HSV1 is present and is active in a high proportion of brains of elderly normal subjects as well as AD patients [31], [39]. However, PKR was activated only in the brain sample from the AD patient. One possible explanation for this is that there might be genetic variability in the PKR activation that could be related to AD development. Recently a genetic association between rs2254985 polymorphism within *EIF2AK2* gene and the risk of AD has been found [28]. Finally it has to be remarked that other stressor factors can contribute to the phosphorylation of PKR in AD patients by the activation of the NF-kß and p38MAPKinase intracellular signalling pathways [55], [56], [57].

**Figure 5:**
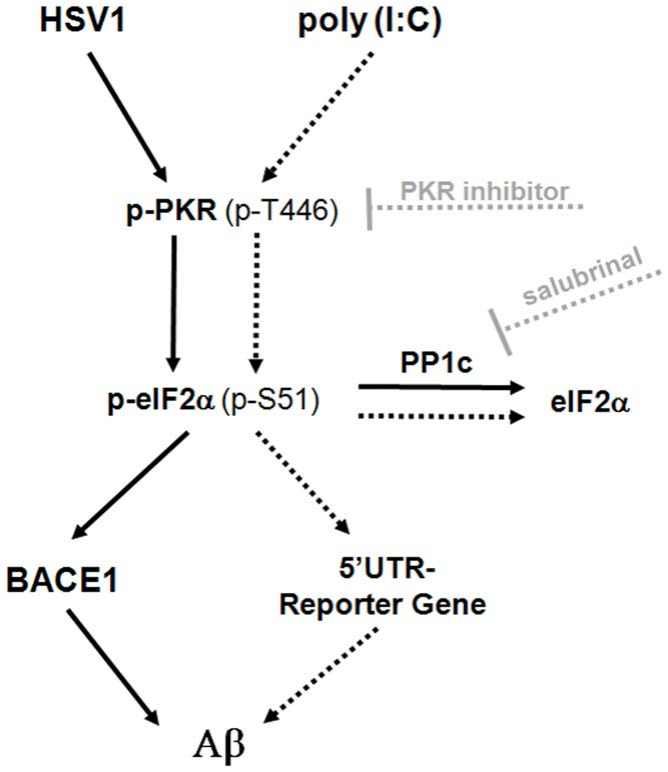
Biochemical pathway linking HSV1 infection and AD. Despite the viral ability to circumvent host defensive mechanisms, including PKR activation, HSV1 activates PKR *in vitro* and *in vivo*. Activated PKR increases eIF2-alpha phosphorylation levels, leading to BACE1 translation de-repression, BACE1 protein up-regulation and Aß production (left track; continuous line). In the right track (dotted line) we present the pharmacological and biological tools that we used to study this pathway: poly (I∶C), to mimic the effect of viral dsRNA; a specific imidazolo-oxindole compound that acts as a potent PKR inhibitor; and a 5′UTR-luc reporter construct used for the evaluation of the translational effect exerted by the PKR-eIF2-alpha pathway over BACE1 5′UTR. Also, we have utilized the PP1c inhibitor salubrinal, which prevents the dephosphorylation of eIF2-alpha.

In conclusion, this study presents further evidence for considering that HSV1 is an aetiological factor contributing to sporadic AD. We confirmed that this neurotropic virus can activate PKR, an eIF2-alpha kinase. Activated PKR results in BACE1 translation and increased Aß production (Fig. 5). Interestingly, despite the fact that a high proportion of elderly people harbour HSV1 DNA in brain, PKR is activated only in brains of AD patients, making this protein a putative therapeutic target in AD. Other features of the life-cycle of the virus and other factors such as APOE genotype might be involved also in the differential pattern of PKR activation observed within HSV1 infected brains.
